# The Effects of Guided Imagery on Heart Rate Variability in Simulated Spaceflight Emergency Tasks Performers

**DOI:** 10.1155/2015/687020

**Published:** 2015-06-07

**Authors:** Zhang Yijing, Du Xiaoping, Liu Fang, Jing Xiaolu, Wu Bin

**Affiliations:** ^1^China Astronaut Research and Training Center, Beijing 100094, China; ^2^National Key Laboratory of Human Factors Engineering, Beijing 100094, China

## Abstract

*Objectives*. The present study aimed to investigate the effects of guided imagery training on heart rate variability in individuals while performing spaceflight emergency tasks.* Materials and Methods*. Twenty-one student subjects were recruited for the experiment and randomly divided into two groups: imagery group (*n* = 11) and control group (*n* = 10). The imagery group received instructor-guided imagery (session 1) and self-guided imagery training (session 2) consecutively, while the control group only received conventional training. Electrocardiograms of the subjects were recorded during their performance of nine spaceflight emergency tasks after imagery training.* Results*. In both of the sessions, the root mean square of successive differences (RMSSD), the standard deviation of all normal NN (SDNN), the proportion of NN50 divided by the total number of NNs (PNN50), the very low frequency (VLF), the low frequency (LF), the high frequency (HF), and the total power (TP) in the imagery group were significantly higher than those in the control group. Moreover, LF/HF of the subjects after instructor-guided imagery training was lower than that after self-guided imagery training.* Conclusions*. Guided imagery was an effective regulator for HRV indices and could be a potential stress countermeasure in performing spaceflight tasks.

## 1. Introduction

Numerous studies and anecdotes showed that astronauts experienced many negative emotions in space, such as anxiety, stress, and depression, which would deteriorate even further under emergency situations and ultimately lead to unreliable or incorrect operations [1]. Some researchers and practitioners have realized that emotion management is an important scientific issue in long-term manned spaceflight missions and many psychology interventions were applied to ground training and in-orbit missions [1, 2]. However, there is limited research investigating the interventions of stress and associated symptoms in Chinese individuals when they perform tasks.

Autonomic cardiac control is crucial in health and social behavior. As a marker of cardiac autonomic nervous system function, heart rate variability (HRV) has been reported to indicate cardiac health, cardiac mortality and morbidity, and overall mortality risk [3]. Cardiac autonomic function through its sympathetic (SNS) and parasympathetic (PNS) branches modulates heart rate (HR). It is also proven to be correlated with physiological effects of depression, anxiety, anger, and stress [4].

As a mind-body relaxation technique, guided imagery has been widely used for decreasing patient's stress and anxiety and increasing an athlete's sport performance [4, 5]. Previous evidences support the effectiveness of guided imagery to relieve stress, anxiety, and depression [4, 6, 7]. Thus, it has been utilized as a treatment strategy in those individuals or patients who need mental and physical relaxation [8, 9]. The use of guided imagery has been reported to reduce self-reported measures of stress, anxiety, and fatigue as well as neuroendocrine measures of stress such as cortisol among nonpregnant subjects [9]. Most of imagery research focuses on sports training and clinical treatment [8, 10]; seldom is it applied to the domain of manned spaceflight [11]. Moreover, among many susceptible physiological biomarkers to evaluate and intervene in mental and physical state, heart rate variability (HRV) is an important clinical and investigational tool to reflect changes in cardiac autonomic regulation under extreme environments [12]. Therefore, guided imagery was applied as a tension countermeasure as well as HRV intervention for Chinese in centrifuge training [11].

HRV is the change in the time interval between heartbeats, from beat to beat. It is controlled by the autonomic nervous system including the sympathetic nervous system (SNS) and the parasympathetic nervous system (PNS). Generally, the SNS activity increases heart rate, and the PNS activity decreases heart rate. HRV is believed to be an indicator of the dynamic interaction and balance between the SNS and the PNS [13, 14]. Thus, HRV is the final result of rhythmic, integrated activity of autonomic neurons and provides a method to measure nervous system competence. Moreover, HRV reflects the capacity of the central autonomic network, including the prefrontal cortex, central nucleus of the amygdala, hypothalamus, and brainstem, to meet and adapt to environmental demands [15] and underpins individual's capacity to regulate their emotions [16] and may be critical to psychological flexibility [17, 18].

Given the high demand of operation pressure in manned spaceflight, the present study aimed to explore the impact of guided imagery training and self-guided imagery training on HRV of Chinese operators when performing spaceflight emergency tasks. This study revealed the enhancing effects of guided imagery on the indices of HRV and identified how instructor-guided imagery plays a more effective role in intervention than self-guided imagery.

## 2. Materials and Methods

### 2.1. Ethics Statement

The study was approved by the ethics commission of China Astronaut Research and Training Center. All subjects signed consent forms prior to participating in the experiment.

### 2.2. Study Design and Subjects

A between-subjects experiment was designed to explore the effects of guided imagery training on physiological variables. The presence of training is a between-subject factor which has two groups: one is imagery group, and the other is control group. For the imagery group, subjects received two kinds of imagery training: instructor-guided and self-guided imagery. On the contrary, subjects in the control group just used the corresponding time to sit still and review the operations. In addition to investigating whether training is effective, another purpose of this study is to investigate if self-guided imagery has the same effect as instructor-guided imagery does. Therefore, both instructor-guided imagery and self-guided imagery belong to the imagery group, but the reference within such group was instructor-guided imagery condition.

The best subjects for this study may be current Chinese astronauts. Unfortunately, it was impossible to have Chinese astronauts participating in this study for the limited number and the free time of them. In the future, some new astronauts will be selected from among scientists and engineers. Besides, most of Chinese astronauts are male. Thus, we recruited twenty-one male students from the astronautics department of Tsinghua University to meet the astronaut selection criterion from the aspect of education background and gender. They were randomly divided into two groups: imagery group (*n* = 11) and control group (*n* = 10). Their ages ranged from 21 to 26 years (Mean = 23.27, SD = 1.60).

Based on previous studies, sleep quality [19, 20] is related to emotion and working performance as well as the function of autonomic nervous system [21]. Therefore, sleep quality was controlled in the experiment. First of all, there were no reports on sleep disturbance among the subjects. Then, the subjects were required to sleep well in the night before experiment, and the experiment was conducted in the working hours. Subjects were also asked to avoid overexcitement activity and drinking alcohol and caffeine 24 hours before participating in the experiment.

The experiment environment factors were maintained the same between the two groups. In the first place, the environment was controlled strictly to make sure the illumination condition is constant (all light on and all curtain closed) and the noise was avoided. Second, the subjects were asked to sit in a chair fixed in the ground and to avoid abrupt and severe body movement. Only forearms and fingers were asked to finish the nine spaceflight operations. These controls generally reduce, if not eliminate, possible artefacts that could influence the HRV analyses.

There are various ways to practice mental imagery. Generally, mental imagery was guided by some instructions given by an experienced psychology expert [8, 11], which was defined in this study as instructor-guided imagery. In addition, we defined self-guided imagery as one way to practice mental imagery without instructions from a trainer. Since the way mental imagery practiced would decide if the image went thoroughly into the mind, the different impacts on physiological indices of the two guided imagery techniques were also investigated in this study. The imagery group practiced instructor-guided imagery and self-guided imagery at two consecutive sessions with intervals of 1 week.

### 2.3. Experiment Process and Intervention

Before the formal experiment, the two groups learned how to perform the nine spaceflight emergency tasks for 90 minutes to make sure the subjects established the correct mental operational model and reach a skill plateau. The nine spaceflight emergency tasks were chosen from the Chinese spaceflight operation handbook. One of the emergency tasks is a procedural activity to handle one malfunction of a spacecraft subsystem. Those activities only induced some minor body movements such as brief reporting, mouse clicking, or putting/loosening the button. The nine tasks appeared in a random sequence for each subject of one group. One subject took about 10 minutes to finish all the nine tasks and called one trail.

The formal experiment included two sessions. In session 1, the imagery group received instructor-guided imagery training, which was conducted by an experienced psychologist. Before guided imagery, the subjects were asked to review the operations. Then, the subjects were led to a quiet room where they lay down on a comfortable chair and closed their eyes. Firstly, the imagery group received intervention instructions and was guided to an image while staying in a welcoming, secure, and comfortable place, to experience some fine things and sceneries through visual, auditory, and body sensation in imagery. The guided words were developed by a Swedish psychological therapist and several clinical psychological experts in Astronaut Center of China. Then the subjects were guided to imagine the operations of the nine tasks. It generally took about half an hour for instructor-guided imagery training. After guided imagery, the subject practiced the nine tasks for two trials. And the physiological data was recorded when subjects were performing the nine spaceflight emergency tasks.

In session 2, self-guided imagery training was conducted by the subjects themselves in accordance with the experience of instructor-guided imagery. After self-guided imagery, the subject practiced the nine tasks for two trials and the physiology data was recorded.

Subjects in the control group also attend two session's experiment. In each session, they practiced the nine tasks for two trials as the imagery group without imagery training. And the physiology data was recorded at the corresponding trials.

### 2.4. Measurements

Self-reports were given by the subjects in imagery group after mental imagery training. The reports were about the effect imagery training had on the subjects.

Electrocardiogram (ECG) was recorded for all subjects by one dynamic multiparametric physiological detector called KF2 (BodyMon Ltd., China). KF2 recorded ECG with 3 leads at a rate of 250 Hz. KF2 was worn around the chest of each subject when performing the nine spaceflight emergency tasks. Disposable silver/silver chloride ECG electrodes were placed at the standard location for ECG recording. And the ECG data were stored on a Secure Digital Card for offline analysis by HRV processing software.

The HRV processing software provides HRV indices in time and frequency domains through the following steps. Firstly, ECG data was automatically corrected firstly using an artefact detection algorithm [22]. Then Fast Fourier Transform (FFT) method was used to calculate spectral power of heart rate variability using a Hanning window with 512 data points. The neighboring windows were overlapping with the 3/4 length of the whole window. Finally, the software generated the HRV results in excel format. The HRV processing software is widely used among Chinese researchers and its reliability and validity have been documented [23–25].

Heart rate variability was analyzed in time and frequency domains [26]. Time domain measurements included heart rate, Standard deviation of all normal NN interval (SDNN), root mean square of successive differences (RMSDD), the percentage of NN interval differences >50 ms from the preceding interval (PNN50), and coefficient of variation of NN interval (CV, calculated by standard deviation of NN intervals divided by mean of NN intervals). SDNN are thought to reflect changes of HRV mediated by both the influence of parasympathetic and sympathetic nerve. RMSSD and PNN50 are thought to reflect the changes of HRV mainly mediated by parasympathetic nerves [27]. Because of the moderate correlation between heart rate and HRV the coefficient of variation (CV) calculation is also reported which attempts to control for differences in heart rate [28].

Frequency domain (spectral) measurement of HRV was obtained by Fast Fourier Transform (FFT) which is one of spectral analysis methods to determine the power at different frequencies and helps to distinguish the contribution of the PNS and SNS to the variability in heart rate. Frequency domain measurements included very low frequency (VLF, 0.0033 to 0.04 Hz), low frequency (LF, 0.04 to 0.15 Hz), and high frequency (HF, 0.15 to 0.4 Hz) power, as well as normalized values (HFNU, LFNU), the total power (TP, 0.0033 to 0.4 Hz), and LF/HF ratio. The LF and HF components are considered as an index of sympathetic and parasympathetic modulation, respectively, and the LF to HF ratio reflects the global sympathovagal balance [3, 29]. Total power represents the total variability in the record. VLF power represents a measure of uncertain value. VLF power may indicate thermoregulation or vasomotor activity, although this has been disputed [30, 31]. It may involve a parasympathetic component [31] and possibly engage the rennin-aldosterone system [31–33].

### 2.5. Statistical Analysis

Stationarity is an important prerequisite in analyzing time series data such as heart rate. Therefore, we checked whether our data is stationary before carrying out the formal analyses. We used the widely used Augmented Dickey-Fuller test which seems to be more suitable as our time series data is quite long (almost 10 minutes, 800–1000 beats); therefore the parameters recommended by Magagnin et al. [34] might not be used properly to our data. The results suggest that the null hypothesis of a unit root (nonstationarity) was all rejected among the 42 RR interval time series samples. Therefore further analyses can be made without many distortions.

HRV indices of each subject were calculated over a short duration of 5 minutes by the HRV processing software. Before the statistical analysis, a homogeneity test of HRV between the imagery and control groups was conducted. The data were acquired from the last two trails of the 90 min practicing period before the formal experiment.

The Shapiro-Wilk test and Levene's test were used to test whether the given HRV data presented with a normal distribution. Two-way repeated measures ANOVA was used to assess the group effect (control group versus imagery group), the effect of training techniques, and interaction between them. All analyses were carried out in SPSS software Release 20 and all statistical tests with a *P* < 0.05 were considered significant. The power of the ANOVA was calculated as reported in this study.

## 3. Results

Homogeneity testing revealed that heart rate variability consisting of SDNN, RMSDD, PNN50, CV, VLF, LF, HF, LF/HF, and TP was equally distributed between the imagery group and control group.

Two-way repeated measures ANOVA was conducted on the HRV indices in the time domain including HR, RMSDD, SDNN, CV, and PNN50. The results showed that RMSDD, SDNN, and PNN50 of imagery group were significantly higher than those of control group (*P* = 0.002, 0.033, and 0.001, resp.; power = 0.900, 0.579, and 0.941, resp.), CV of imagery group was marginally significantly higher than that of control group (*P* = 0.061, power = 0.470), and HR of two groups has no statistical significance (*P* = 0.106, power = 0.364). The interaction effect of imagery training and training technique was not significant. Moreover, analysis of HR, RMSDD, SDNN, CV, and PNN50 showed that there was no statistical significance between the two techniques.  Table 1 gives a summary of HRV indices in the time domain of the control group and imagery group with the two techniques.

Two-way repeated measures ANOVA was conducted on the HRV indices in frequency including VLF, LF, HF, LF/HF, TP, LFNU, and HFNU. The results showed that the effect of imagery training on the VLF, LF, HF, and TP was significant (*P* = 0.011, 0.044, and 0.005, resp.; power = 0.741, 0.529, and 0.827, resp.), and VLF, LF, HF, and TP of imagery group were higher than those of control group. The effect of the training technique on LF/HF was marginally significant (*P* = 0.085, power = 0.407); LF/HF of the subjects after instructor-guided imagery training was lower than that after self-guided imagery training.  Table 2 gives a summary of HRV indices in frequency domain of the control group and imagery group with the two techniques.

Moreover, the imagery group received a subjective survey about the training effects. Participants of the imagery group listed several benefits they had received from guided imagery training, such as the following: “helped me feel confident in operation,” “it helped me deal with stress better,” and “I feel clearer about the operation procedure than before.” 10 of 11 subjects in the imagery group reported no perceived barriers with instructor-guided imagery training. On the other hand, 9 of 11 thought that they were unable to enter a peaceful virtual-environment when they performed self-imagery training. But self-imagery training provided a chance for them to review the operations and therefore improve their skills. Overall, the subjects in imagery group thought that guided imagery provided an emotional support to them and released the operation tension.

## 4. Discussion

### 4.1. Study Outcomes

The purpose of this study was to investigate the effects of guided imagery on biologic measures of stress and effort in Chinese operators performing spaceflight emergency tasks.

In this study, HRV of the imagery and control groups before imagery training were at the same level and had no statistical difference. After guided imagery training, there was a statistically significant increase in RMSDD, SDNN, PNN50, VLF, HF, LF, and TP as well as marginally significant increase in CV in imagery group compared to that in control group. HR, LF/HF, and LFNU of imagery group decreased nonsignificantly compared with control group. HRV is a commonly used tool for assessing autonomic function of the heart [3], as well as stress [4, 35] and fatigue [36]. Previous studies demonstrated that HRV is significantly lower in cancer patients than the healthy population [37] and decreased HRV was associated with significantly shorter survival rates in cancer patients [38]. In the present study, SDNN and LF were improved with 2 weeks of guided imagery training, indicating that autonomic function of the heart was improved by guided imagery. From the current data, we can conclude that the training is at least effective during the 10-minute operations before which the astronauts were asked to do such imagery work either instructor-guided or self-guided.

The guided imagery uses verbal suggestions to create a flow of thoughts that focus the individual's attention on imagined visual, auditory, tactile, or olfactory sensations. This method can reduce the activity of autonomic nervous systems and increase the activity of parasympathetic nervous system both directly and indirectly. First, it directly regulates the respiratory rhythms and refocuses people's attention from environmental and persona stress to imagined stimuli which would lead to reduced arousal and increased relaxation [39]. Second, guided imagery may indirectly influence the two systems by improving the motor control skills of the subjects as the stimulated mental rehearsal of desired movements was indeed very similar to the brain activity that would accompany the actual motor movement [40, 41]. The combined effect can shift autonomic nervous system function toward increased parasympathetic nervous system (PNS) and reduced sympathetic nervous system (SNS) activity [42]. As suggested by a graded sympathetic activation protocol by Porta et al. [43], it means that guided imagery can result in an augmentation of the high frequency power of HRV, PNN50, and RMSSD [27], and HF power reflects HRV attributable to respiratory sinus arrhythmia and is regarded as a marker of vagal modulation of R-R intervals and a cardiac parasympathetic effect [3, 44]. It will also increase the total power (TP) of HRV as well as SDNN (total power and SDNN which is the measure of total heart rate variability reflecting both the parasympathetic and sympathetic cardiac modulation) [3].

These findings were supported by previous researches [8, 45] and added evidence to the effectiveness of guided imagery to reduce stress while performing spaceflight tasks. However, there is no clear explanation of how guided imagery brings about positive physical changes; one possible explanation has been provided by Bedford, who believes that perceptual processes are involved in imagery [46]. For this study, the imagery training combined six minutes of imagery relaxation and mind rehearsal of nine operations, and the result may contribute to two aspects. Firstly, as previous research proved, mental imagery relieved the tension of the operator, reducing the stress and anxiety caused by performing tasks and time pressure; therefore, HRV showed a rise in the imagery group in this study. This point was proved by other researches. Kong explored the effects of guided imagery intervention among coronary artery patients and found that SDNN, TP, and HF were increased [47]. Chuang et al. applied music therapy to breast cancer patients and reported that SDNN, TP, HF, and LF increased [48]. Secondly, the visual rehearsal is intended to train the subjects' minds and create the neural patterns in our brain by teaching the muscles how to operate [49]. This in-mind practice works on improving the operator's performance [10] and therefore decreases the operator effort and workload. As Aasman and Li pointed out that HRV indices were regarded as the representative of operator effort or mental workload [50–52], the increase of HRV indices in this study represented the decrease of the operator workload.

Compared with instructor-guided imagery, self-guided imagery showed a little different impact on HRV in the imagery group. LF in the self-guided imagery session was significantly higher than that in instructor-guided imagery. According to self-reports of the subjects, self-guided imagery could not lead them to a peaceful state but they could mentally visualize what they should do while performing the nine tasks. A possible explanation for this finding may be related to the combined effect of imagery in this study. A previous study concluded that imagery was related to focused breathing, relaxation, stress, and stress-related symptoms, as well as mental rehearsal [53]; thus, it would be no surprise that the stress induced by emergency conditions was counteracted by the effort or workload decrease with the increase of operational skill in session 2. This result implied that guided imagery had an immediate effect on decreasing operation stress, which potentially may impact mental workload and ultimately operation performance.

Heart rate variability (HRV) is a marker of cardiac autonomic nervous system function and has been presented as a good tool to study physiological effects of work-related stress [18, 54] and work-related worries [55]. A number of studies and reviews (e.g., [21, 54]) have indicated that HRV is reduced in patients with depression and anxiety, even without cardiovascular disease. While studies have often focused on links between decreased HRV, negative emotions, and poor physical health, increased HRV is related to well-being [56] and reductions in negative affect [57–61].

### 4.2. Implications and Limitations

Guided imagery is a simple, easily taught, and acquired method, but it has rarely been implemented as a training method for spaceflight operations. It was typically used as a psychological intervention tool before a manned spaceflight mission or applied to astronauts when some negative emotional symptoms appear. Plessinger recommended that athlete training incorporates mental imagery along with physical practice [10]. Not only can mental imagery improve specific motor skills but also it seems to enhance motivation, mental toughness, and confidence, all of which will help elevate the performance level. The study went further, combining guided imagery with spaceflight operation training. But it should be additionally tested and researched to find the effect mechanism of guided imagery and the appropriate program to combine guided imagery and operation training.

It should be noted that this study has some limitations. First of all, small population size is a continuous worrisome issue for researchers like us whose job is to train a special group of operators. As performing the tasks in our research requires a complex simulation system which is quite costly to use, it is very difficult to use a large size of subjects. Specifically, subjects in this study were all students from the same university excluding the Chinese astronauts. Therefore, some of the results will be tested further in astronaut training and other experiments. Moreover, the effect of mental imagery is limited by the engagement of subjects, which was reported by subjective reports from subjects. Further investigation requires the combination of electroencephalogram (EEG) and other physiological indicators to evaluate the engagement of subjects and thus to objectively analyze the role of mental imagery and the mechanism itself. In addition, virtual reality technology can be used to design mental imagery training programs to improve the effect of imagery training.

## 5. Conclusions

The results of this study showed that guided imagery training significantly increased HRV indices in Chinese subjects while performing spaceflight emergency tasks. It implied that guided imagery training is one effective way to decrease stress and therefore improve operation performance. From the current data, we can conclude that the training is at least effective during the 10-minute operations before which the astronauts were asked to do such imagery work either instructor-guided or self-guided. As concluded from this study, guided imagery training seems promising and beneficial to ensure astronauts well-being and stable performance in orbit. Hence, a program with operation practice combined with imagery training was recommended for astronaut operation training. In practice, self-guided imagery was applied to the astronauts in Shenzhou Nine and Shenzhou Ten before they conducted Manually Rendezvous and Docking. The effect of imagery was committed by the astronauts.

## Figures and Tables

**Table 1:** Summary of HRV indices in time domain of control group and imagery group with the two techniques.

Time domain HRV	Training techniques	Control group	Imagery group	*P* value of training technique	*P* value of imagery training	*P* value of interaction effect

HR (count/min)	IGI	78.82 ± 3.22	76.60 ± 3.02	0.813	0.106	0.344
	SGI	82.64 ± 3.03	74.30 ± 3.46			

RMSDD (ms)	IGI	33.54 ± 2.32	45.10 ± 5.50	0.935	0.002	0.255
	SGI	27.82 ± 2.19	51.70 ± 9.10			

SDNN (ms)	IGI	59.03 ± 3.34	83.89 ± 16.08	0.579	0.033	0.623
	SGI	58.45 ± 5.80	74.22 ± 7.64			

CV	IGI	0.077 ± 0.004	0.102 ± 0.017	0.564	0.061	0.440
	SGI	0.078 ± 0.007	0.089 ± 0.007			

PNN50/%	IGI	12.75 ± 3.02	24.13 ± 4.37	0.962	0.001	0.364
	SGI	8.67 ± 1.83	27.80 ± 6.66			

Note: (1) standard errors are shown in parentheses;

(2) IGI was short for instructor-guided imagery, and SGI was short for self-guided imagery.

**Table 2:** Summary of HRV indices in frequency domain of control group and imagery group with the two techniques.

Indices	Training techniques	Control group	Imagery group	*P* value of training technique	*P* value of imagery training	*P* value of interaction effect
VLF	IGI	473.1 ± 102.8	876.1 ± 171.8	0.279	0.011	0.676
	SGI	594.3 ± 150.8	1147.7 ± 266.8			

LF	IGI	491.0 ± 41.6	707.9 ± 118.4	0.540	0.044	0.715
	SGI	522.6 ± 92.5	832.6 ± 209.9			

HF	IGI	243.5 ± 26.9	421.9 ± 109.1	0.603	0.005	0.241
	SGI	176.9 ± 26.3	593.3 ± 174.5			

LF/HF	IGI	2.18 ± 0.26	2.00 ± 0.26	0.085	0.473	0.787
	SGI	3.00 ± 0.33	2.60 ± 0.65			

TP	IGI	1207.4 ± 112.9	2005.9 ± 304.0	0.334	0.004	0.477
	SGI	1294.1 ± 190.6	2573.8 ± 585.4			

LFNU	IGI	67.09 ± 2.10	65.40 ± 2.56	0.505	0.126	0.295
	SGI	72.91 ± 3.10	64.10 ± 5.11			

HFNU	IGI	32.91 ± 2.10	34.60 ± 2.56	0.505	0.126	0.295
	SGI	27.09 ± 3.10	35.90 ± 5.11			

Note: (1) standard errors are shown in parentheses;

(2) IGI was short for instructor-guided imagery, and SGI was short for self-guided imagery.
